# A Supramolecular Approach for Enhanced Antibacterial Activity and Extended Shelf-life of Fluoroquinolone Drugs with Cucurbit[7]uril

**DOI:** 10.1038/s41598-018-32312-6

**Published:** 2018-09-17

**Authors:** Hamdy S. El-Sheshtawy, Suchandra Chatterjee, Khaleel I. Assaf, Meenakshi N. Shinde, Werner M. Nau, Jyotirmayee Mohanty

**Affiliations:** 1 Institute of Nanoscience & Nanotechnology, Kafrelsheikh University, 33516 Kafrelsheikh, Egypt; 2 Chemistry Department, Faculty of Science, Kafrelsheikh University, 33516 Kafrelsheikh, Egypt; 3 Food Technology Division, Bhabha Atomic Research Centre, Mumbai, 400 085 India; 4 Department of Life Sciences and Chemistry, Jacobs University Bremen, Campus Ring 1, 28759 Bremen, Germany; 5 Radiation & Photochemistry Division, Bhabha Atomic Research Centre, Mumbai, 400 085 India; 6 Homi Bhabha National Institute, Training School Complex, Anushaktinagar, Mumbai, 400 094 India

## Abstract

The host-guest interactions of a third-generation fluoroquinone, danofloxacin (DOFL), with the macrocyclic host cucurbit[7]uril (CB7) have been investigated at different pH values (~3.5, 7.5, and 10). The photophysical properties have been positively affected, that is, the fluorescence yield and lifetime increased, as well as the photostability of DOFL improved in the presence of CB7. The antibacterial activity of DOFL is enhanced in the presence of CB7, as tested against four pathogenic bacteria; highest activity has been found towards *B*. *cereus* and *E*. *coli*, and lower activity towards *S*. *aureus* and *S*. *typhi*. The antibacterial activity of two additional second-generation fluoroquinones, i.e., norfloxacin and ofloxacin, has also been investigated in the absence as well as the presence of CB7 and compared with that of DOFL. In case of all drugs, the minimum inhibitory concentration (MIC) was reduced 3–5 fold in the presence of CB7. The extended shelf-life (antibacterial activity over time) of the fluoroquinone drugs in the presence of CB7, irrespective of four types of bacteria, can be attributed to the enhanced photostability of their CB7 complexes, which can act as better antibiotics with a longer expiry date than uncomplexed DOFL.

## Introduction

Fluoroquinolones (FQs), derived from nalidixic acid, constitute one of the most successful classes of antibiotic drugs in therapeutic applications that are used in the treatment of a variety of bacterial infections^1,2^. The ‘ideal’ fluoroquinolone combines good clinical efficacy with low minimal inhibitory concentration (MIC) without any cytotoxicity. To achieve low MIC values, different generations of fluoroquinolones have been developed over the years as their substitution pattern modulates their antimicrobial activity^1,2^, such as the insertion of a fluorine atom at position 6 and a piperazine ring^3^. FQs bear both an acidic group (carboxylic acid) and a basic tertiary amino one, inferring amphoteric properties. Depending on pH, FQs prevail in their protonated (below pH 6), neutral or zwitter ionic (pH 6–8), or anionic forms (above pH 8)^4,5^. It has been established that the zwitter ionic form of FQ is responsible for antimicrobial activity as this form is sufficiently lipid-soluble to be able to penetrate tissues^6^. However, over the past 20 years, fluoroquinolone research has aimed at improving activity against Gram-positive microorganisms, whilst retaining its bioactivity against Gram-negative organisms. Thus, first-generation quinolones are active against Gram-negative microorganisms, second-generation ones such as norfloxacin and ofloxacin are active against Gram-negative and some Gram-positive microorganisms, while third- and fourth-generation quinolones have an expanded activity against Gram-positive microorganisms^3^. Besides the covalent/synthetic modification of the basic chemical structure, noncovalently linked, externally controlled supramolecular host-guest interactions using macrocyclic hosts such as cyclodextrins, cucurbiturils, etc.^7,8^ are promising to improve and control the antibacterial activity of drugs. In a recent study, Henriques *et al*. reported that gallic acid shows good antibacterial activity upon complexation with β-cyclodextrin and its derivatives^9^. Zhang *et al*.^10,11^ reported an enhanced antibacterial activity of a porphyrin photosensitizer, and Bai *et al*. described a supramolecular antibiotic switch to regulate the antibacterial activity using cucurbit[7]uril (CB7) as macrocyclic host^12^. Zhang *et al*. have also designed bacterial responsive supramolecular complex of perylene diimide derivative for photothermal therapy with high selectivity towards facultative anaerobic bacteria^13^. Recently, cucurbit[*n*]urils (CB*n*) have received attention due to the strong interactions of their negatively charged carbonyl portals with positively charged organic dye molecules, metal cations, nanoparticles, proteins, and surfactants or through peripheral binding with polyanions such as polyoxometalates^14–25^. Among the CB*n* homologues, CB7 is the most prominent one; it is highly water soluble, forms the strongest inclusion complexes with organic guests, and improves drastically their molecular properties such as photostability, aqueous solubility, fluorescence behavior, etc.^7,8,18,26–30^. In recent years, cucurbituril-based host-guest complexes are found to have potential applications in nanosensors to discriminate cancer cells^31^, supramolecular assays for drug detection^32^, storage/delivery of polysulfide in lithium-sulfur batteries^33^, white light emitting materials^34^, molecular switching^35^, supramolecular catalysis to modulate the activity of reaction intermediates^22,36^ and stimuli-responsive supramolecular assemblies^25^/vesicles for targeted drug delivery^37^ etc.

Danofloxacin (DOFL) is a third-generation fluoroquinolone antimicrobial drug with a rapid bactericidal activity against a broad range of pathogens responsible for a number of disease syndromes of economic importance in the commercial rearing of livestock^38^. Though DOFL is used in veterinary medicine as the mesylate salt for the treatment of respiratory diseases in cattle, swine, and chicken, there is a major concern to human health for causing toxicity, allergy, and bacterial resistance problems^6^. Hence, there is a need either to remove DOFL from cow milk or to lower drug intake through more effective antibacterial activity in the treatment of animal disease. In a recent study, Valcárcel *et al*. have shown that β-cyclodextrin-modified nanocellulose can be used for the selective fluorimetric determination and extraction of danofloxacin from milk samples^39^. In this article, we establish a supramolecular approach to enhance antibacterial activity and shelf-life of CB7-encapsulated DOFL against two Gram positive (*Staphylococcus aureus: S*. *aures; Bacillus cereus: B*. *cerus*) and two Gram negative (*Escherichia coli: E*. *coli; Salmonella typhi: S*. *typhi*) pathogenic bacteria with 3–5 fold reduced MIC. The extended shelf-life is attributed to an increased photochemical and thermal stability of the drug in the presence of CB7.

## Results

**Figure 1:**
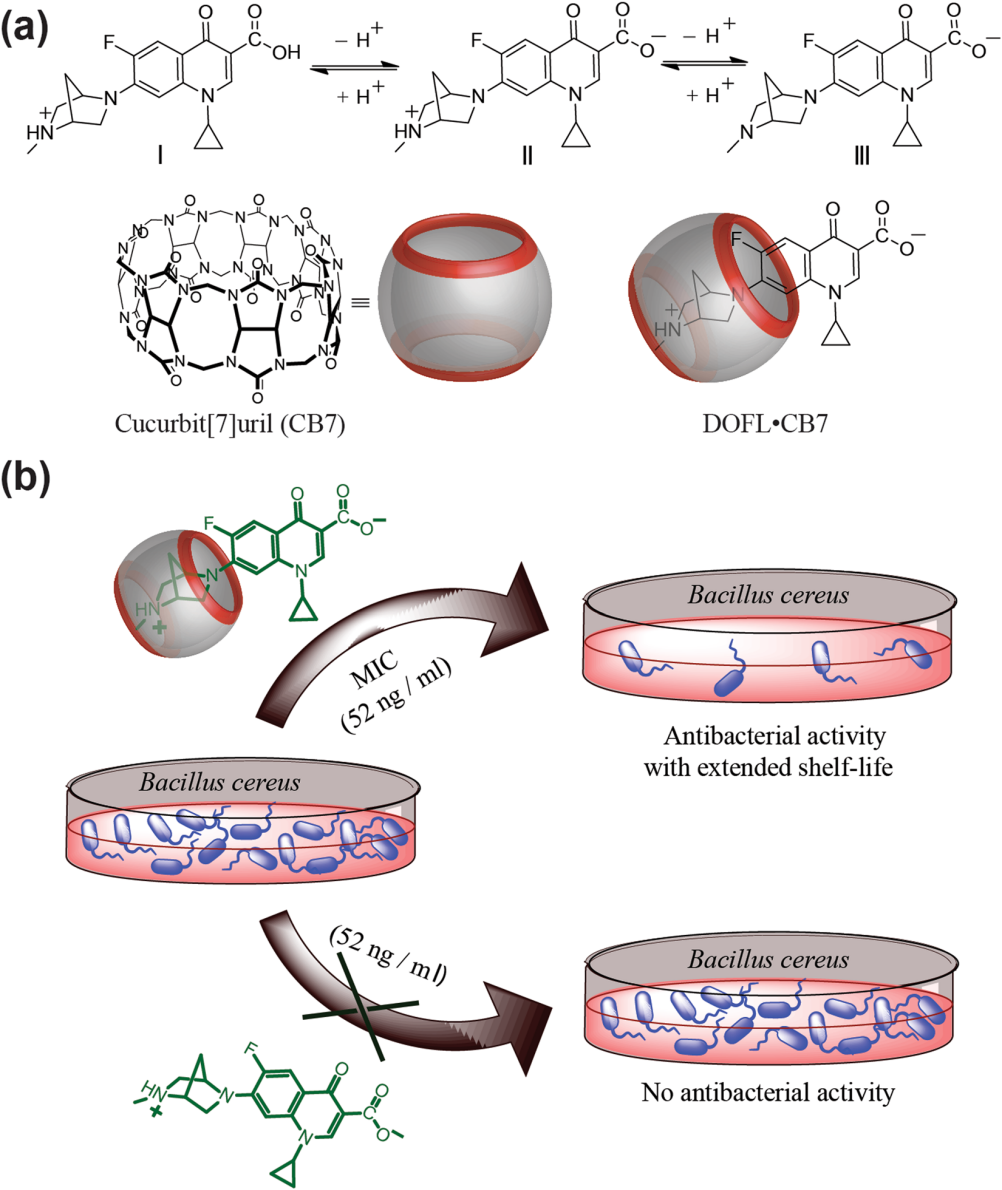
Chemical structures & schematic depiction. (**a**,**b**) Chemical structures of different forms of DOFL and CB7. (**a**) Schematic representation of antibacterial activity of DOFL (52 ng/ml) in the absence and presence of CB7 (**b**).

Depending on the pH of the solution, danofloxacin exists in three different forms (cationic (I), DOFLH_2_^+^; zwitter ion (II), DOFLH, and anionic (III), DOFL^−^; Fig. 1a); the zwitter ionic form can also tautomerize into a putative neutral form in which the carboxylic group is stabilized by forming a six-membered cyclic structure through intramolecular hydrogen bonding^40^. The changes in the absorbance by varying pH of the drug at a particular wavelength showed two inflection points, corresponding to two different p*K*_a_ values (inset of Fig. S1, Supporting Information). The ground-state p*K*_a_ values evaluated from the fitted data (*cf*. solid curve in the inset of Fig. S1) were 6.3 ± 0.1 and 8.6 ± 0.1, which corresponded to the prototropic equilibria of the deprotonation of the carboxylic OH (p*K*_a1_)^41^ and the tertiary 1,4-diazabicyclo[2.2.1]heptyl methylamino group (p*K*_a2_), respectively^42^.

### Absorption and Fluorescence Behavior of Danofloxacin with CB7

**Figure 2:**
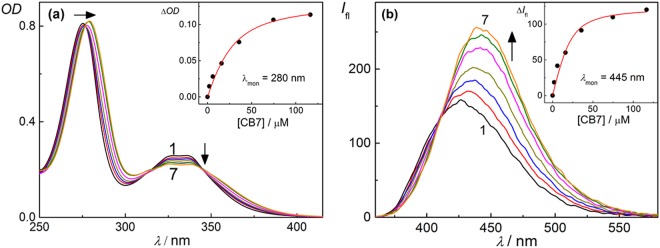
Absorption and fluorescence spectral studies. (**a**,**b**) Absorption (**a**) and fluorescence (**b**) spectra of DOFL (20 μM) at pH 7.5 at different concentrations of CB7. [CB7]/μM: (1) 0.0, (2) 2.0, (3) 6.0, (4) 16.0, (5) 35.7, (6) 74.6 and (7) 116.5. The respective absorption and fluorescence titration curves are shown in the insets; the solid line represents the fitted curve according to a 1:1 complexation model.

Considering the multiple p*K*_a_ values of DOFL, we have investigated the binding interaction of the different prototropic forms of DOFL with CB7 at three pH values, 3.5, 7.5, and ~10. At pH 7.5, DOFL exists as DOFLH, which showed an absorption within the range of 250–425 nm with an intense narrow band at ~275 nm and a weaker broad band at 340 nm^40^. Upon addition of CB7, there was a bathochromic shift in the 275 nm band and a slight increase in the absorbance of the broad band along with three isosbestic points (Fig. 2a). This major change in the absorption spectra of DOFL clearly indicated a strong interaction with CB7. However, there was no significant change in the absorption spectra of DOFLH_2_^+^ and DOFL^−^, prevalent at lower and higher pH values, upon the addition of CB7.

On the emission front, DOFLH at pH 7.5 showed a broad emission with spectral maximum at 425 nm. Upon gradual addition of CB7, the fluorescence intensity increased along with a bathochromic shift of ~17 nm and an isoemissive point at ~410 nm, indicating the formation of a host-guest complex (Fig. 2b). At pH ~3.5, DOFLH_2_^+^ showed a broad fluorescence band with peak position at ~442 nm, whereas at pH ~10.2, DOFL^−^ showed an emission maximum at ~433 nm. In contrast to the titrations of DOFLH, the fluorescence intensity of DOFLH_2_^+^ and DOFL^−^ decreased upon addition of CB7 to the solution. DOFLH_2_^+^ showed a bathochromic shift (~3 nm) at the peak position and DOFL^−^ displayed a hypsochromic shift of ~8 nm with an isoemissive point at 408 nm (Fig. S2). The characteristic changes in the fluorescence quantum yield (*Φ*_f_) and lifetime (*τ*_f_) of all three forms suggested host-guest complex formation with CB7. Coumarin 1 in water was used as the standard to measure the fluorescence quantum yields of all forms of the drug with and without CB7 in water^43^. The combined photophysical parameters of all three forms of danofloxacin in water, with and without CB7, are listed in Table 1.

**Table 1:** Photophysical parameters and binding constant values of DOFL with CB7 at different pH values.

System	pH	λ_em_/nm	Φ_f_	τ/ns (%)	*K*/(M^−1^) (Fluorescence)	*K*/(M^−1^) (ITC)
DOFL	3.5	444	0.65	7.5		
DOFL-CB7		447	0.47	7.8	(2.1 ± 0.2) ×10^4^	(2.1 ± 0.2) ×10^5^
DOFL	7.5	425	0.27	2.3 (22) 7.5 (78)		
DOFL-CB7		442	0.43	4.8 (44) 8.7 (56)	(1.6 ± 0.9) ×10^5^	(1.7 ± 0.1) ×10^5^
DOFL	10.2	433	0.10	2.4		
DOFL-CB7		425	0.07	2.2 (89) 7.1 (11)	(6.5 ± 0.5) ×10^3^	(7.3 ± 0.5) ×10^3^

### Binding Constants of CB7•DOFL Systems from Fluorescence and ITC Measurements

**Figure 3:**
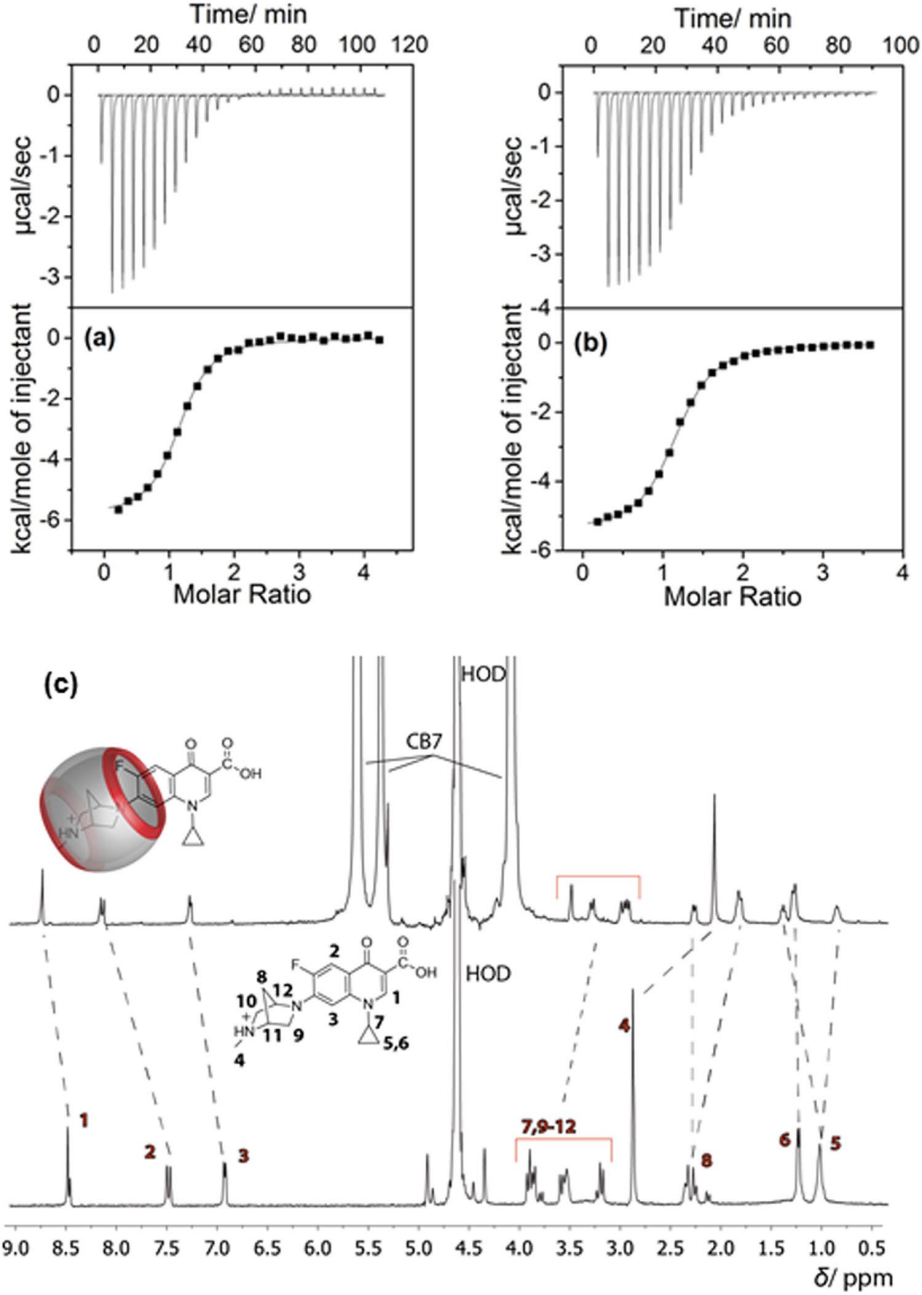
Isothermal titration calorimetric and ^1^H-NMR studies. (**a**–**c**) ITC isotherms for titration of DOFL guest with CB7 at 25 °C in aqueous solution at pH 3.5 (**a**) and pH 7.5 (**b**). The top panel shows the instrumental power function *versus* time (injected aliquots) plot. The lower panel shows the plot for heat of reaction obtained from the integration of the calorimetric traces, plotted against the host/guest molar ratio. (**c**) ^1^H-NMR spectra (400 MHz) of DOFL in the absence (lower panel) and presence (upper panel) of CB7 in D_2_O at pD ~3.

The binding constants for the three different forms with CB7 were determined based on the fluorescence titrations by using a 1:1 binding model^44–46^. The binding constants obtained from the fluorescence titration curves (Insets of Fig. 2 and Insets of Fig. S2) reached a maximum for the CB7•DOFLH complex (1.6 × 10^5^ M^−1^) and were lower for both, CB7•DOFLH_2_^+^ (2.1 × 10^4^ M^−1^) and CB7•DOFL^−^ (6.5 × 10^3^ M^−1^), see Table 1. Due to the small changes in the photophysical properties of DOFLH_2_^+^ and DOFL^−^ upon addition of CB7, the binding constants for all three complexes were more reliably determined by using isothermal titration calorimetry (ITC), which confirmed the ability of CB7 to complex DOFL at different pH values (Fig. 3a,b). The binding constants of DOFL to CB7 at pH 7.5 and 10.2 agree well with the ones obtained by using fluorescence titrations. However, the ITC results at pH 3.5, revealed a higher binding constant for the CB7•DOFLH_2_^+^ complex compared to the fluorescence titration. A net 1:1 complexation enthalpy (Δ*H* = −5.9 kcal mol^−1^) for CB7•DOFLH_2_^+^ and (Δ*H* = −5.4 kcal mol^−1^) for CB7•DOFLH and entropy (−*T*Δ*S* = 1.4 kcal mol^−1^) for CB7•DOFLH_2_^+^ and (−*T*Δ*S* = 1.7 kcal mol^−1^) for CB7•DOFLH were obtained from ITC measurements. Due to the low binding constant of the CB7•DOFL^−^ complex, we focused on the CB7•DOFLH and CB7•DOFLH_2_^+^ complexes in the subsequent investigations.

### ^1^H-NMR and ^19^F-NMR Measurements

To establish the binding sites of the drug molecules with the CB7 host, we carried out ^1^H-NMR measurements at pD ~3; the shifts in the NMR signals are shown in Fig. 3c. Three aromatic proton signals of DOFL (labelled as 1, 2 and 3) were downfield shifted (Δ*δ* ranges from 0.3 to 0.7 ppm) in the presence of CB7 due to deshielding of the nuclei, which suggested that these protons reside near to the carbonyl portals of CB7. In contrast, the aliphatic protons (4 and 7–12) displayed upfield shifts (Δ*δ* ranges from 0.2 to 0.8 ppm), which is attributed to the encapsulation of the diazabicyclo[2.2.1]heptyl group into the hydrophobic cavity of CB7. An upfield shift of the F atom by 1.2 ppm in the ^19^F-NMR signal also confirms the inclusion of the fluorine atom in the cavity^47^.

### Energy Optimized Structures of CB7•DOFL Systems

Dispersion-corrected quantum-chemical calculations (DFT, wB97XD/6-31G*) were performed to determine the structure of the host-guest complexes and their stability. The optimized structure for all complexes showed that the diazabicyclo[2.2.1]heptyl group is encapsulated inside the CB7 cavity. For CB7•DOFLH_2_^+^ and CB7•DOFLH, the complexes were stabilized over the CB7•DOFL^−^ complex through ion-dipole interactions (see SI, Fig. S3). The calculated binding energies followed the order: CB7•DOFLH_2_^+^ > CB7•DOFLH > CB7•DOFL^−^.

### Determination of p*K*_a_ Values of CB7•DOFL Systems

**Figure 4:**
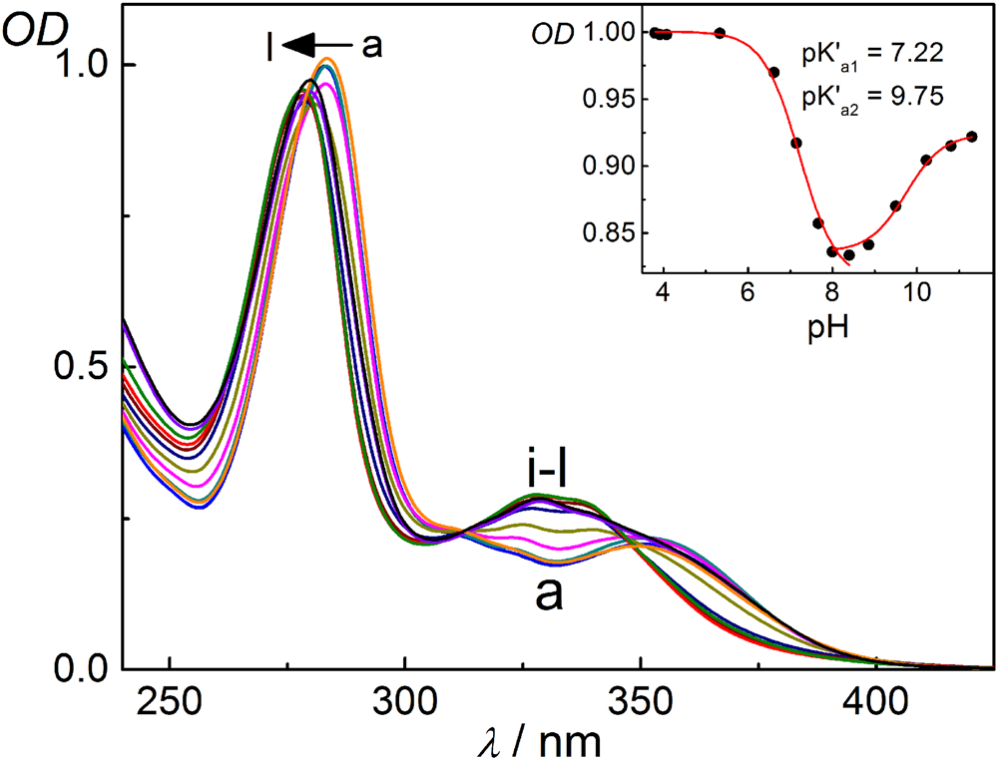
p*K*_a_ studies. Absorption spectra of DOFL (~22 μM) in the presence of 1 mM CB7 at different pH values: (**a**) 2.7, (**b**) 3.8, (**c**) 4.1, (**d**) 5.3, (**e**) 6.6, (**f**) 7.1, (**g**) 7.7, (**h**) 8.0, (**i**) 8.4, (**j**) 8.9, (**k**) 10.2 and (**l**) 11.3. The inset shows the variation in *OD* with pH at 285 nm.

Others and we have established that there is a substantial variation in the p*K*_a_ values of the guest molecules upon inclusion complex formation with macrocycles^28,44,48–52^. Whenever the conjugate acid shows stronger binding interaction with the macrocyclic host than its corresponding base, the p*K*_a_ value usually exhibits a large upward shift^52^. In the current complex system, due to the existence of similar kind of binding interactions, an upward shift of both the p*K*_a_ values of DOFL in the presence of CB7 is observed. The consequence of complexation on the prototropic equilibria of DOFL was studied by monitoring the variations in the absorption spectra of the drug at different pH values, with 1 mM of CB7 and the results are shown in Fig. 4. In this system, assuming the complexation of all the three forms of drug with CB7, the acid-base equilibria of the drug should follow a six-state thermodynamic cycle^48^, as shown in Scheme S1, SI. Excess concentration of CB7 was used to confirm virtually the complete binding of the three different prototropic forms of the drug with CB7.

As becomes evident in Fig. 4, the spectral changes are significant at ~285 nm in the investigated pH range from 3.5–12. The pH titration curve corresponding to the first deprotonation equilibrium for the carboxylic OH of the fluoroquinolone unit in the CB7 complex presented a value of p*K*′_a1_ = 7.22 ± 0.05, about one unit higher than the p*K*_a1_ value of the uncomplexed drug. Such a shift in the p*K*_a_ values specifies that the CB7-complexed drug converts into a stronger base, by ~12 times, than its uncomplexed form. The pH titration curve at higher pH values corresponds to the dissociation constant of the tertiary diazabicyclo[2.2.1]heptyl methylamino group in its CB7 complex; its p*K*′_a2_ value was found to be 9.75 ± 0.1, considerably higher than the p*K*_a2_ value for the uncomplexed drug (p*K*_a2_ = 8.6 ± 0.1).

### Antibacterial Activity and Photostability of CB7•DOFL Systems

**Figure 5:**
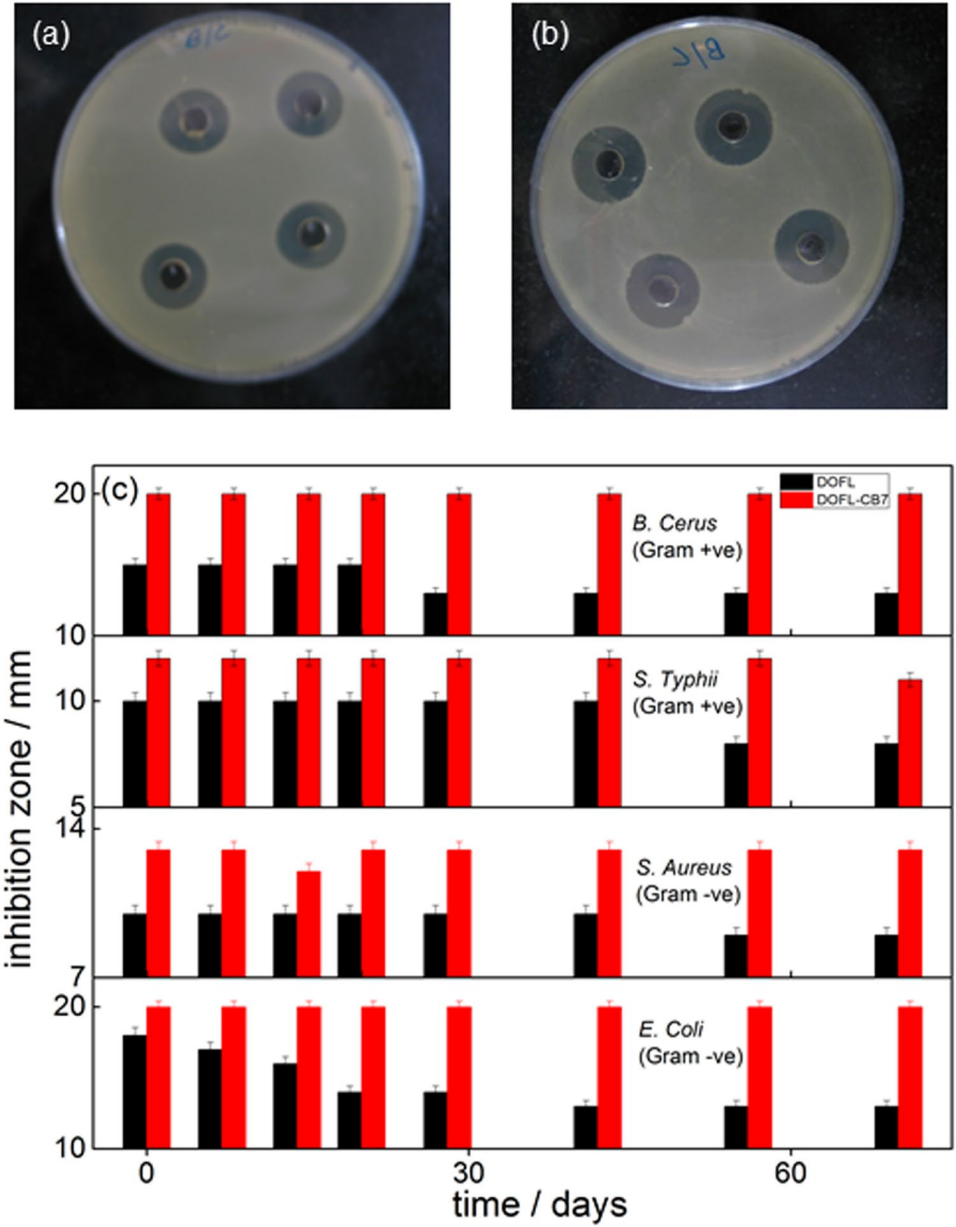
Antibacterial activity and shelf-life studies. (**a**,**b**) Images of the inhibition zones of the bacterial growth of *B*. *cereus* in the presence of DOFLH without (**a**) and with (**b**) CB7 at pH 7.5. (**c**) Bar chart representation of antibacterial activity (in terms of inhibition zone) of DOFLH in the absence (black bar) and presence (red bar) of CB7 with time against four bacteria.

It is reported that the zwitterionic form of fluoroquinolones is mainly responsible for their antibacterial activity^6^. Since CB7 showed a high binding affinity towards this most active form, we studied the effect of CB7 complexation on the antibacterial activity of DOFL. In detail, we measured the reduction of bacterial growth of two Gram positive (*Staphylococcus aureus* and *Bacillus cereus*) and two Gram negative (*Escherichia coli* and *Salmonella typhi*) pathogenic bacteria by DOFL with and without CB7 at pH 3.5, 7.5 (physiological pH region), and 8.1. The radial diameters of the inhibition zone are presented in Tables 2 and S1. The images of the inhibition zone of the bacterial (*B*. *cereus*) growth in the presence of free and CB7-complexed DOFL at pH 7.5 are displayed in Fig. 5a,b, respectively. The CB7-complexed zwitterionic form (pH ~7.5) showed a significant antibacterial activity against *B*. *cereus* (Gram +ve) and *E*. *coli* (Gram −ve) with a 20-mm inhibition zone as compared to the 15-mm inhibition zone of the uncomplexed form in these two bacteria. Moreover, the complexed zwitterionic form showed a maximum activity (20 mm inhibition zone) against *B*. *cereus* (Gram +ve) and *E*. *coli* and the least activity (12–13 mm) was recorded in *S*. *aureus* (Gram +ve) and *S*. *typhi* (Gram −ve) (Tables 2 and S1). Though the antibacterial activity of DOFLH_2_^+^ (at pH 3.5) was lower than that of the zwitterionic form (at pH 7.5), the activity of CB7-complexed cationic form of DOFLH_2_^+^ followed a similar trend as in the complexed zwitterionic form – irrespective of the individual micro-organism (Tables 2 and S1). At pH 8.1, where almost all the complexed DOFLH is in the zwitterionic form, the antibacterial activity also followed a similar trend and was found to be maximum for CB7-DOFLH system as compared to those at pH 3.5 and 7.5 (Table 2 and Fig S4, SI). It may be noted here that even though the upward p*K*_a_ shift effectively brings down the zwitterionic concentration by ~30% at pH 7.5, the increased contribution of the CB7-complexed protonated form cumulatively enhances the antibacterial activity. Since our experiments are related to bacterial growth in the physiological condition, we have carried out all the other experiments at pH 7.5. Furthermore, the minimum inhibitory concentration (MIC) of DOFLH with and without CB7 towards these four pathogenic bacteria was determined which matches the reported value^37^, see Table S2. Expectedly, the higher inhibitory effect of the drug in the presence of CB7 translated into a (medicinally desirable) decrease of the minimal inhibitory concentration (MIC), e.g. from ~0.261 μg/ml against *B*. *cereus* in the absence of CB7 to 0.052 μg/ml in the presence of 10 μM CB7 (Table S2) and is pictorially represented in Fig. 1b.

**Table 2:** Antibacterial activity (in terms of inhibition zone) of DOFL (~14 μM) with and without CB7 (1 mM) towards four pathogenic micro-organisms at two different pH values.

pH	System	Zone of inhibition/mm			
		*B*. *cereus* (Gram +ve)	*S*. *aureus* (Gram +ve)	*S*. *typhi* (Gram −ve)	*E*. *coli* (Gram −ve)
8.1	DOFL	18 ± 0.3	21 ± 0.2	24 ± 0.2	22 ± 0.4
	DOFL/CB7	20 ± 0.4	24 ± 0.4	26 ± 0.3	27 ± 0.3
7.5	DOFL	16 ± 0.5	9.5 ± 0.4	10 ± 0.4	15 ± 0.5
	DOFL/CB7	20 ± 0.5	12 ± 0.4	13 ± 0.4	20 ± 0.5
3.5	DOFL	12 ± 0.5	6 ± 0.4	10 ± 0.5	12 ± 0.5
	DOFL/CB7	15 ± 0.5	9 ± 0.4	15 ± 0.5	16 ± 0.5

Antibacterial activity of a drug molecule generally follows two mechanisms, either bacteriostatic or bactericidal. Bacteriostatic drugs only prevent the growth of bacteria while bactericidal ones permanently kill the bacteria^38^. In our system, it has been observed that CB7-complexed DOFL showed a higher antibacterial activity, but the activity remained bacteriostatic in nature.

**Figure 6:**
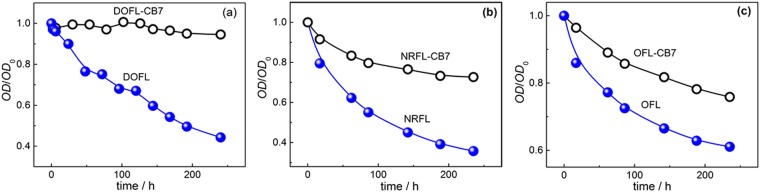
Photostability studies. (**a**–**c**) The changes in absorbance at the respective peak positions with time of DOFL (**a**) NRFL (**b**) and OFL (**c**) in the absence and presence of CB7, at ambient conditions at pH 7.5.

Furthermore, we investigated the shelf-life (activity/quality over a specified period of time) of pre-dissolved danofloxacin solutions with and without CB7 at pH 7.5 stored under ambient conditions. Figure 5c presents the inhibition zone/bacterial activity of uncomplexed and CB7-complexed DOFL in the four studied bacteria with time. For CB7-complexed DOFL, the antibacterial activity remained constant for more than two months, irrespective of bacteria type. In contrast, uncomplexed DOFL showed a 30–40% decrease in antibacterial activity over the same period in all types of bacteria used in this study. We attribute the increased shelf life in the presence of CB7 to a reduced photochemical or thermal degradation of the drug. Indeed, when we followed sample integrity by absorbance and fluorescence, we observed a strong degradation for DOFL solutions kept at ambient conditions for a period of 10 days, which could be virtually suppressed in the presence of CB7 (Figs 6a and S5a, SI). Similarly, thermal degradation at elevated temperature was effectively suppressed in the presence of CB7 as shown in the absorbance changes monitored at 60 °C for about 2 hrs (Fig. S6a, SI).

To generalize, we also investigated the host-guest interactions of two second-generation fluoroquinolones (ofloxacin: OFL and norfloxacin: NRFL) with CB7 by monitoring their absorption and fluorescence changes (Figs S7 and S8, SI) and chemical shift in the NMR signals (upfield shift for the aliphatic protons and downfield shift for the aromatic protons as shown in Fig. S9, SI) as well as their antibacterial activity. Unlike DOFL, both NRFL and OFL contain a piperazine instead of the diazabicyclo[2.2.1]heptyl group. As observed in DOFL, CB7-complexed OFL and NRFL showed higher antibacterial activity than the uncomplexed drug at both selected pH conditions, 3.5 and 7.5 (Fig. S10 and Table S1, SI). However, NRFL remained inactive against *B*. *cereus* and *E*. *coli*, regardless of the presence of CB7. The MIC value of the free and CB7-complexed NRFL and OFL drugs towards these pathogens are listed in Table S2. The extended shelf-life of the two drugs against the above bacteria in the absence and presence of CB7 at pH 7.5 was also determined, see Fig. S10, SI. In line with the observed increase in biological activity in the presence of CB7, the photo/thermal degradation of NRFL and OFL samples, directly followed by absorbance or fluorescence, was again reduced in both cases (Figs 6b,c and S5b,c and S6b,c), although the effect was less pronounced than for DOFL (see above). The combined studies suggest that the stabilizing and efficacy-enhancing properties of CB7 formulations on fluoroquinolone drugs are not specific for a single compound.

From a chemical reactivity point of view, the extended shelf-life of the fluoroquinolone drugs in the presence of CB7 is presumably due to a stabilization of the aromatic or bicyclic amino substituents within the CB7 cavity. Norfloxacin and ofloxacin, for example, are known to undergo photochemical or thermal degradation of the piperazine side chain, either by degradation to an amino group or oxidation^40^.

## Discussion

CB7 forms host-guest complexes with the three prototropic forms of a third generation fluoroquinolone derivative, danofloxacin (DOFL). The fluorescence quantum yield, excited-state lifetime, and photostability of DOFL, in their distinct prototropic forms, are modulated significantly in the presence of CB7. Similarly, complexation behaviour is observed for two additional second generation fluoroquinolone drugs, namely, norfloxacin (NRFL) and ofloxacin (OFL). The observed shifts towards higher p*K*_a_ values for the drugs encapsulated by CB7 is consistent with the better stabilization of the protonated forms of the guests by more attractive (for deprotonation of cationic guests to their neutral forms) or less repulsive (for deprotonation of neutral guests to their anionic forms) interactions with the carbonyl portals of the host^48,52^.

In regard to potential applications, the antibacterial activity of all three drugs is considerably enhanced in the presence of CB7, as we explored against four pathogenic bacteria at both, pH 3.5 and 7.5. Among the different variants studied, DOFL displays highest activity towards *B*. *cereus* and *E*. *coli* and lowest activity towards *S*. *aureus* and *S*. *typhi* at both pH values. Furthermore, the substantial reduction in MIC value (3–5 fold) and extended shelf-life along with increased antibacterial efficacy, generalized for all three drugs, are highly encouraging for the use of CB7 for the design and development of new long-acting antibiotic formulations.

## Methods

### Absorption and fluorescence measurements

Absorption spectra of the samples were recorded from solutions in 1-cm quartz cuvettes on a Varian Cary 4000 UV/Vis spectrophotometer or a Jasco UV–Vis spectrophotometer (model V-650). Steady-state fluorescence spectra were recorded by using a Varian Eclipse or a Hitachi F-4500 fluorometer. The fluorescence lifetime measurements at room temperature were performed by time-correlated single-photon-counting (TCSPC) on an FLS-920 fluorometer (Edinburgh instrument) incorporating a pulsed diode laser (PDL 800-B from Picoquant, λ_ex_ = 373 nm, FWHM *ca*. 50 ps) suitable for lifetime measurements down to 300 ps. The fluorescence decays could be satisfactorily fitted (χ^2^ < 1.05) by using mono/bi-exponential decay functions.

### ITC measurements

Isothermal titration calorimetry experiments were carried out on a VP-ITC from Microcal, Inc., at 25 °C. The binding equilibria were studied using a cellular guest (danofloxacin) concentration of 0.12 mM, to which a 20–30 times more concentrated host (CB7) solution was titrated. Typically, 27 consecutive injections of 10 μL were used. All solutions were degassed prior to titration. Heats of dilution were determined by titration of host (CB7) solution into water. The first data point was removed from the data set prior to curve fitting with Origin 7.0 software according to a one-set-of-sites model. The knowledge of the complex stability constant (*K*_a_) and molar reaction enthalpy (Δ*H*°) enabled the calculation of the standard free energy (Δ*G*°) and entropy changes (Δ*S*°) according to Δ*G*° = −RT ln *K*_a_ = Δ*H*° − *T*Δ*S*°.

### ^1^H- and ^19^F-NMR measurements

^1^H-NMR and ^19^F-NMR measurements were carried out in D_2_O (99.8%). The signals were recorded using a JEOL ECX spectrometer (400 MHz).

### Photo- and thermal stability measurements

The photobleaching of FQ drugs with and without CB7 was monitored by measuring the absorbance/fluorescence at different times with daylight irradiation (ambient conditions) at pH 7.5. Thermal stability of these drugs was also observed by monitoring the absorbance changes of the drugs at 60 °C at different times.

### Antibacterial activity measurements

Antibacterial assay was carried out by using the agar well diffusion method. Bacterial strains *S*. *aureus* (MU-50), *B*. *cerus* (NCIM-2156), *E*. *coli* (M-16), *and S*. *typhi* (SL1344), were used as indicator for this analysis. The antibacterial activity and the minimum inhibitory concentration (MIC) in terms of inhibition zone of these bacterial strains were determined by the standard agar diffusion method as per NCCLS; in short, cultures were grown overnight in nutrient broth. The cultures were diluted with saline to obtain an inocula of 10^7^ CFU/ml using 0.5 McFarland’s Standard and immediately plated. Wells were bored on the plate by using a cork borer (8 mm) and different dilutions of the drug samples were added to the wells and subsequently incubated at 37 °C for 24 hrs. The zone of inhibition values were estimated by measuring the diameter of the inhibition zone against test micro-organisms. The lowest dilution showing a significant zone of inhibition was considered as MIC. All the experiments were carried out in quadruplicate.

### DFT-calculations

Quantum-chemical calculations were performed in gas-phase with the Gaussian 09 package, utilizing dispersion-corrected density functional theory (wB97XD) in combination with a 6–31G* basis set.

## Electronic supplementary material

Supplementary Information
