# Supplementary material for: A Supramolecular Approach for Enhanced Antibacterial Activity and Extended Shelf-life of Fluoroquinolone Drugs with Cucurbit[7]uril
Source: Sci Rep. 2018 Sep 17;8:13925. doi: 10.1038/s41598-018-32312-6 (PMC6141578; doi:10.1038/s41598-018-32312-6)
Supplement: Supplementary file 1 — Supplementary Information [file 41598_2018_32312_MOESM1_ESM.pdf]

Supporting Information

A Supramolecular Approach for Enhanced Antibacterial Activity and Extended Shelf-life of Fluoroquinolone Drugs with Cucurbit[7]uril

Hamdy S. El-Sheshtawy,[1,2] Suchandra Chatterjee,[3] Khaleel I. Assaf,[4] Meenakshi N. Shinde,[5] Werner M. Nau,*[4] and Jyotirmayee Mohanty*[5,6]

[1] Institute of Nanoscience & Nanotechnology, [2]Chemistry Department, Kafrelsheikh University, 33516 Kafrelsheikh, Egypt

[2] Food Technology Division, Bhabha Atomic Research Centre, Mumbai 400 085, India

[3] Department of Life Sciences and Chemistry, Jacobs University Bremen, Campus Ring 1, 28759 Bremen, Germany

[4] Radiation & Photochemistry Division, Bhabha Atomic Research Centre, Mumbai 400 085, India

[5] Homi Bhabha National Institute, Training School Complex, Anushaktinagar, Mumbai 400094, India

Experimental Section

Materials used

Danofloxacin, ofloxacin and norfloxacin were obtained from Sigma-Aldrich and used as received. CB7 was synthesized according to the reported modified procedure[1-3]. Nanopure water (Millipore Gradiant A10 System; conductivity of 0.06 µS cm^{-1}) was used throughout for solution preparation. The pH of the solution was adjusted by adding dilute perchloric acid or sodium hydroxide solution, and was measured using a pH meter model PC 2700 from EUTECH instruments, India. In the calculation of the CB7 concentration, a correction for the water content (15 wt%) was always considered.

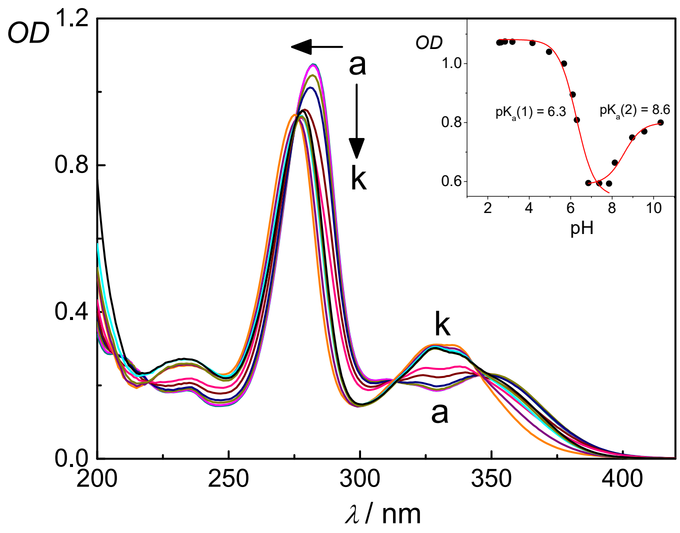

Figure S1. Absorption spectra of DOFL (20 μM) at different pH values: a) 2.7, b) 3.8, c) 4.1, d) 5.3, e) 6.6, f) 7.1, g) 7.7, h) 8.0, i) 8.9, j) 10.2 and k) 11.3. Inset shows the variation in OD with pH at 285 nm.

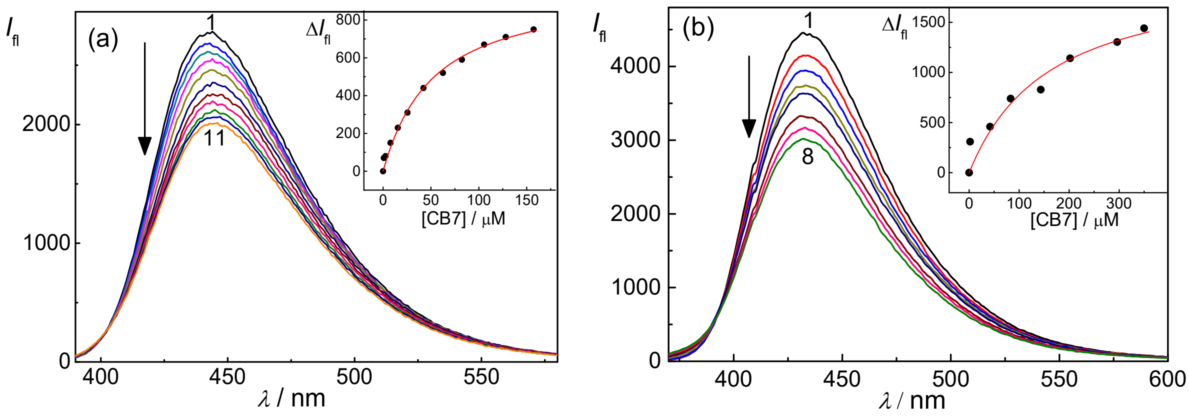

Figure S2. Fluorescence spectra of DOFL (~10 μM) at pH 3.5 (A) and pH 10.2 (B) at different concentrations of CB7. (a) [CB7]/μM: (1) 0.0, (2) 2.6, (3) 7.7, (4) 15.4, (5) 25.6, (6) 42.2, (7) 62.5, (8) 82.4, (9) 105.6, (10) 128.4 and (11) 157.3 and (b) [CB7]/ μM: (1) 0, (2) 2, (3) 6, (4) 83, (5) 143, (6) 202, (7) 296, and (8) 350. λ_{ex} = 315 nm. Insets: The respective fluorescence titration curves of $DOFLH_2^+$ and $DOFL^-$ in the presence of CB7 (●). The solid line represents the fitted curve corresponding to 1:1 complex formation.

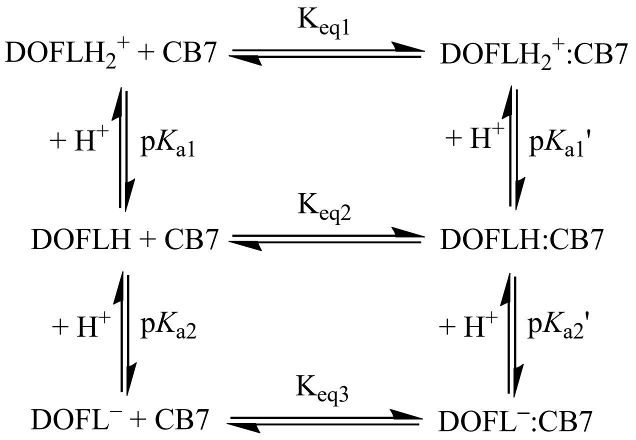

Scheme S1. Six-state model.

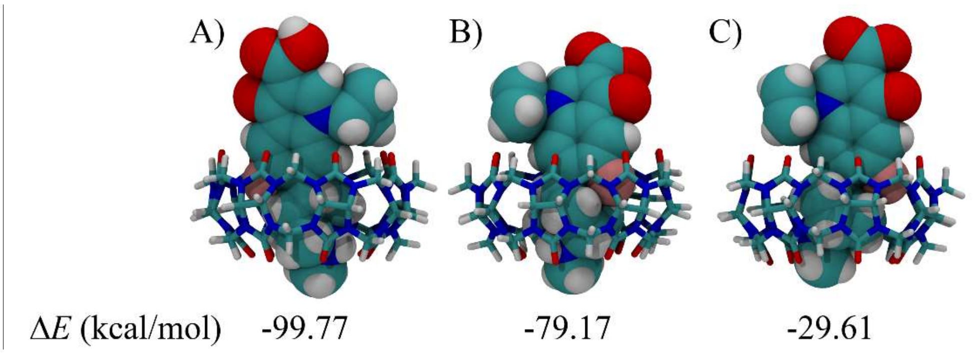

Figure S3. DFT-optimized structures (with the wB97xd/6-31G* method in Gaussian 09[4]) of CB7• DOFL complexes in three different forms (cationic (A), $DOFLH_2^+$; zwitterionic (B), DOFLH, and anionic (C), $DOFL^-$). Calculated binding energies are given below the structures in kcal/mol.

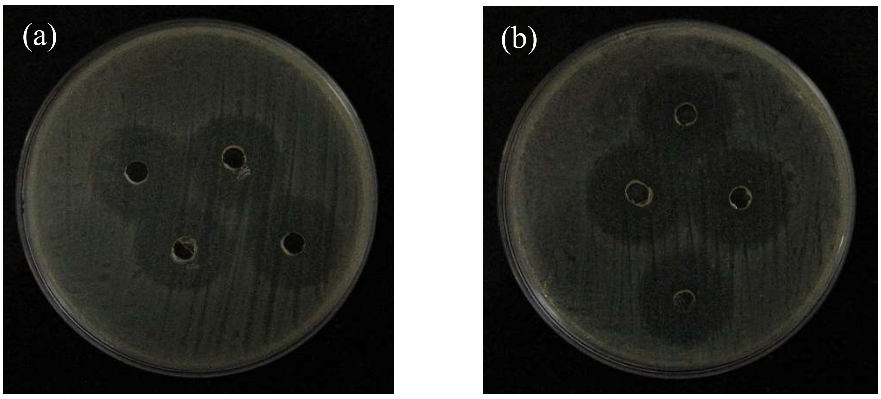

Figure S4. Images of the inhibition zones of the bacterial growth of *E. coli* in the presence of DOFLH without **(a)** and with **(b)** CB7 at pH 8.1.

Table S1. Antibacterial activity (in terms of inhibition zone) of danofloxacin (DOFL), norfloxacin (NRFL) and ofloxacin (OFL) with and without CB7 (1 mM) towards four pathogenic micro-organisms at two different pH values.

pH	Sample	Zone of inhibition/ mm			
		B. cereus (Gram +ve)	*S. aureus* (Gram +ve)	*S. typhii* (Gram -ve)	*E. coli* (Gram -ve)
7.5	DOFL	16 ± 0.5	9.5 ± 0.4	10 ± 0.4	15 ± 0.5
	DOFL/CB7	20 ± 0.5	12 ± 0.4	13 ± 0.4	20 ± 0.5
	NRFL	0	8 ± 0.4	6 ± 0.4	0
	NRFL/CB7	0	10 ± 0.4	10 ± 0.4	0
	OFL	15 ± 0.5	8 ± 0.4	8 ± 0.4	16 ± 0.5
	OFL/CB7	18 ± 0.5	10 ± 0.4	12 ± 0.4	18 ± 0.5
3.5	DOFL	12 ± 0.5	6 ± 0.4	10 ± 0.5	12 ± 0.5
	DOFL/CB7	15 ± 0.5	9 ± 0.4	15 ± 0.5	16 ± 0.5
	NRFL	0	5 ± 0.4	5 ± 0.4	0
	NRFL/CB7	0	7 ± 0.4	8 ± 0.4	0
	OFL	11 ± 0.5	5 ± 0.4	6 ± 0.4	11 ± 0.5
	OFL/CB7	15 ± 0.5	7 ± 0.4	8 ± 0.4	14 ± 0.5

Table S2. Minimal inhibitory concentration of danofloxacin (DOFL), norfloxacin (NRFL) and ofloxacin (OFL) with and without CB7 (10-20 μM) towards four pathogenic micro-organisms at two different pH values.

pH	Sample	Minimal inhibitory concentration (μg/ml)			
		B. cereus (Gram +ve)	*S. aureus* (Gram +ve)	*S. typhii* (Gram -ve)	*E. coli* (Gram -ve)
7.5	DOFL	0.261	0.174	0.174	0.261
	DOFL/CB7	0.052	0.087	0.075	0.065
	NRFL	-	0.146	0.146	-
	NRFL/CB7	-	0.087	0.073	-
	OFL	0.102	0.170	0.170	0.170
	OFL/CB7	0.064	0.073	0.064	0.085
3.5	DOFL	0.261	0.261	0.218	0.261
	DOFL/CB7	0.065	0.104	0.087	0.075
	NRFL	-	0.218	0.218	-
	NRFL/CB7	-	0.073	0.087	-
	OFL	0.170	0.254	0.254	0.254
	OFL/CB7	0.073	0.085	0.073	0.085

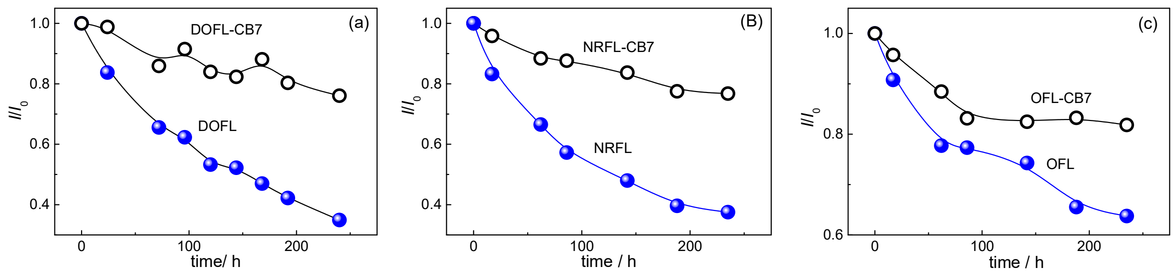

Figure S5. The changes in fluorescence intensity at the respective peak positions with time of DOFL **(a)**, NRFL **(b)** and OFL **(c)** in the absence and presence of CB7 at ambient conditions at pH 7.5.

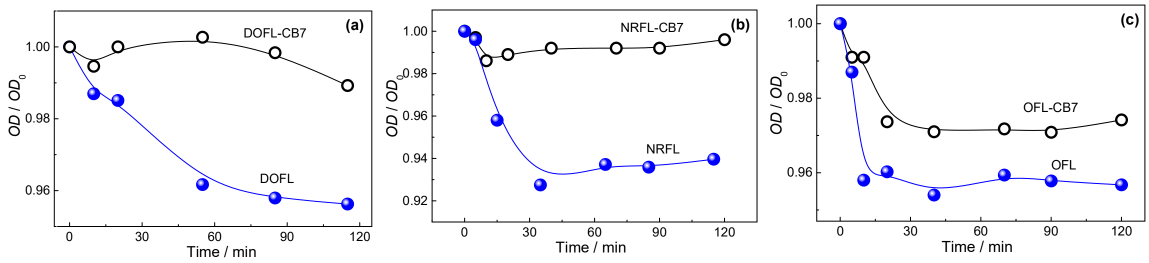

Figure S6. The changes in optical density at the respective peak positions with time of DOFL **(a)**, NRFL **(b)** and OFL **(c)** in the absence and presence of CB7 at 60 °C at pH 7.5.

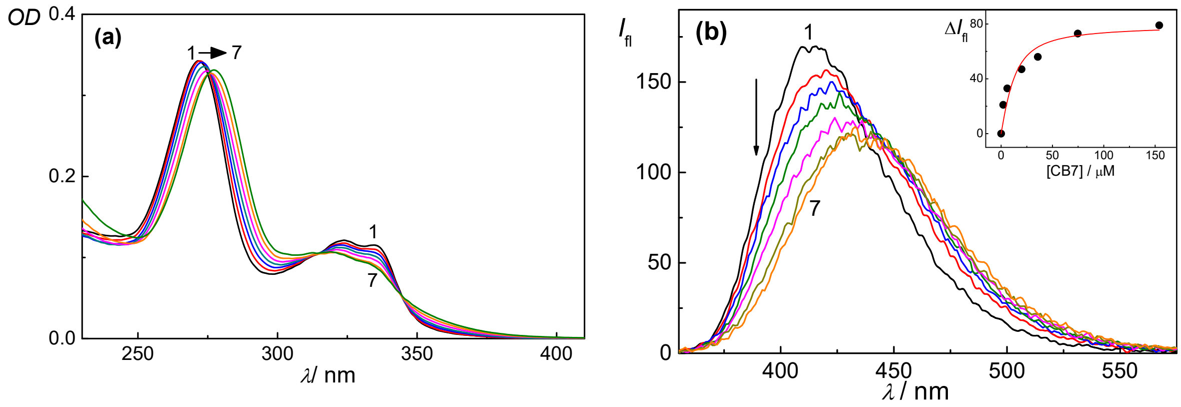

Figure S7. Absorption **(a)** and fluorescence (**b**) spectra of NRFL (10 μM) at pH 7.7 at different concentrations of CB7. [CB7]/μM: (1) 0.0, (2) 2.0, (3) 6.0, (4) 16.0, (5) 35.7, (6) 74.6 and (7) 153.8. Inset shows the fluorescence titration curve; the solid line represents the fitted curve according to a 1:1 complexation model. λ_{ex} = 343 nm. Binding constant (K_a) value evaluated from binding curve is (1.4 ± 0.8) × 10^5 M^{-1}.

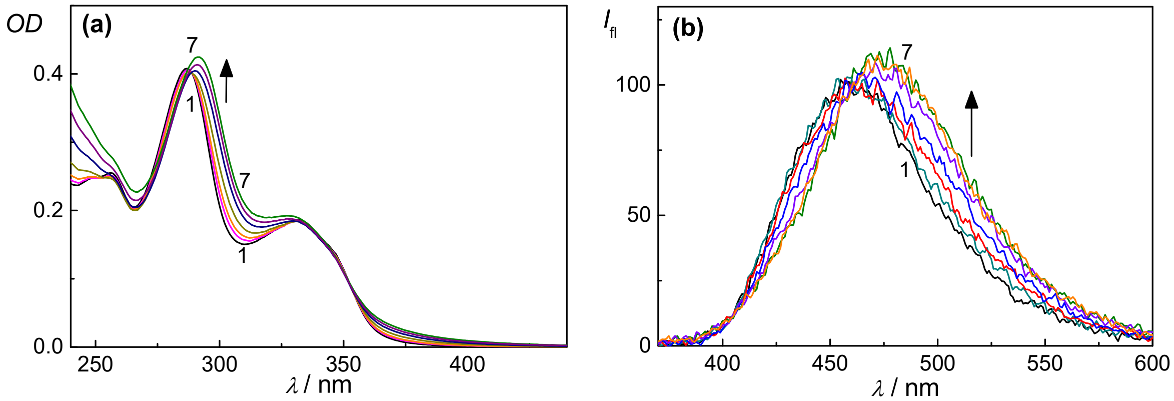

Figure S8. Absorption **(a)** and fluorescence (**b**) spectra of OFL (20 μM) at pH 7.6 at different concentrations of CB7. [CB7]/μM: (1) 0.0, (2) 17.5, (3) 82.0, (4) 185.4, (5) 378.0, (6) 553.8 and (7) 715.0.

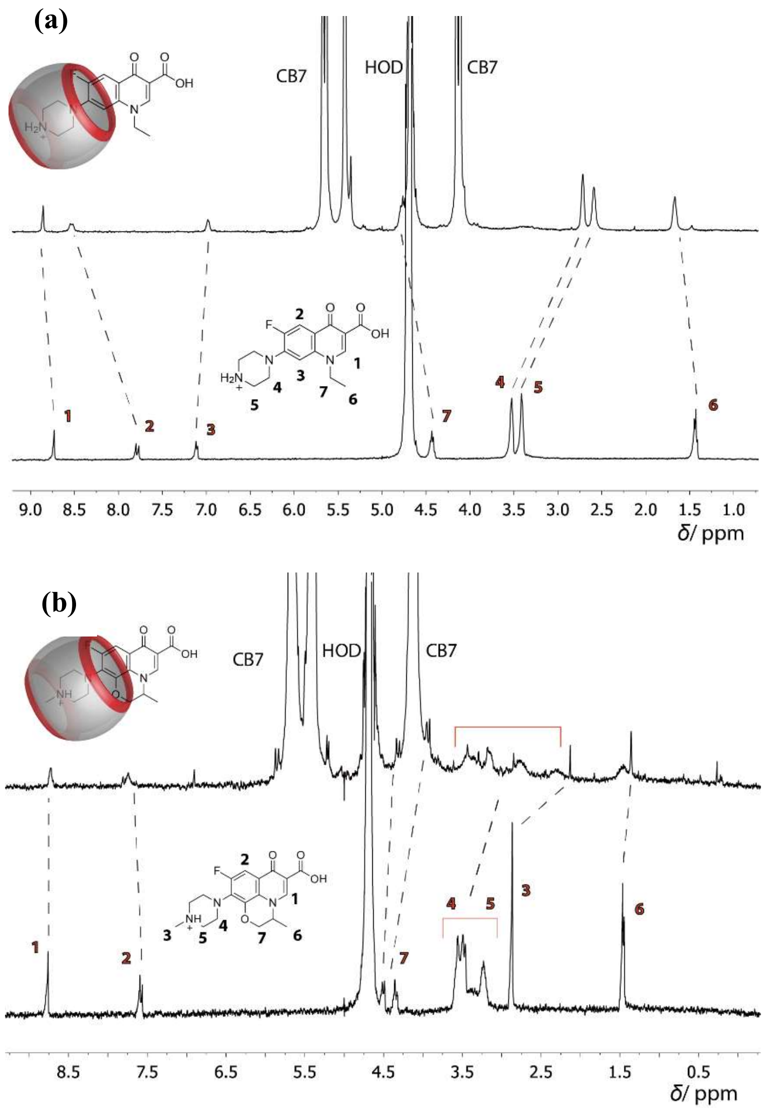

Figure S9. ^{1}H-NMR spectra (400 MHz) of NRFL (**a**) and OFL (**b**) in the absence (lower panel) and presence (upper panel) of CB7 in D_2O at pD ~3.

(a)

(b)

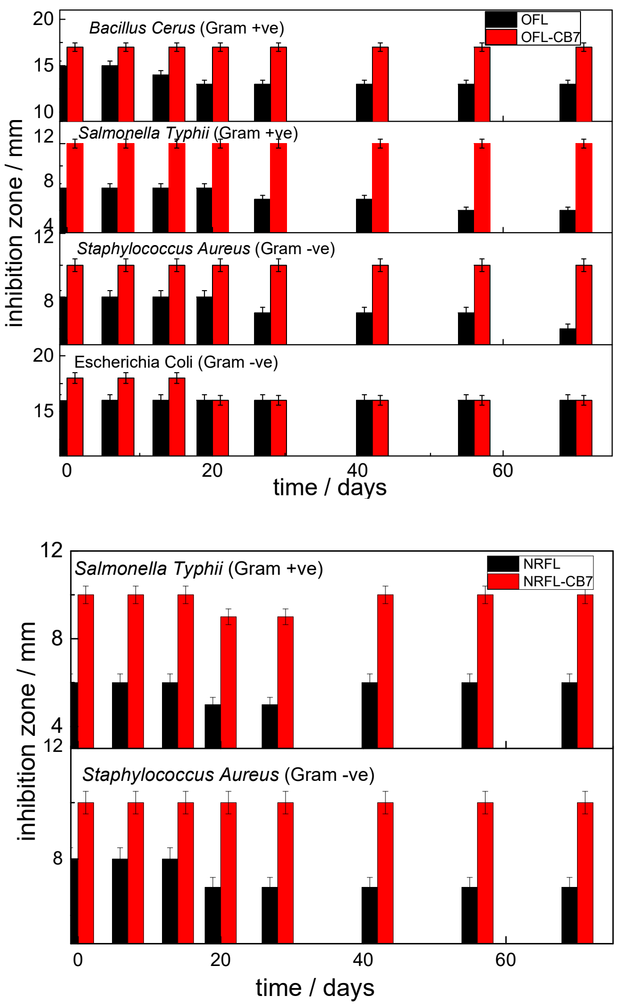

Figure S10. Bar chart representation of the antibacterial activity (in terms of inhibition zone) of OFL **(a)** in the absence (black bar) and presence (red bar) of CB7 with time showing against all four bacteria and NRFL **(b)** in the absence (black bar) and presence (red bar) of CB7 with time showing against two bacteria at pH 7.5.

References

1. Marquez, C., Huang, F. & Nau, W.M. Cucurbiturils: Molecular nanocapsules for time-resolved fluorescence-based assays. *IEEE Trans. NanoBiosci.* **3**, 39-45 (2004).

2. Kim, J. et al. New cucurbituril homologues: Syntheses, isolation, characterization, and X-ray crystal structures of cucurbit[*n*]uril (*n* = 5, 7, and 8). *J. Am. Chem. Soc.* **122**, 540-541 (2000).

3. Day, A., Arnold, A.P., Blanch, R.J. & Snushall, B. Controlling factors in the synthesis of cucurbituril and its homologues. *J. Org. Chem.* **66**, 8094-8100 (2001).

4. Gaussian 09, M. J. Frisch, G. W. Trucks, H. B. Schlegel, G. E. Scuseria, M. A. Robb, J. R. Cheeseman, G. Scalmani, V. Barone, B. Mennucci, G. A. Petersson, H. Nakatsuji, M. Caricato, X. Li, H. P. Hratchian, A. F. Izmaylov, J. Bloino, G. Zheng, J. L. Sonnenberg, M. Hada, M. Ehara, K. Toyota, R. Fukuda, J. Hasegawa, M. Ishida, T. Nakajima, Y. Honda, O. Kitao, H. Nakai, T. Vreven, J. A. Montgomery, Jr., J. E. Peralta, F. Ogliaro, M. Bearpark, J. J. Heyd, E. Brothers, K. N. Kudin, V. N. Staroverov, T. Keith, R. Kobayashi, J. Normand, K. Raghavachari, A. Rendell, J. C. Burant, S. S. Iyengar, J. Tomasi, M. Cossi, N. Rega, J. M. Millam, M. Klene, J. E. Knox, J. B. Cross, V. Bakken, C. Adamo, J. Jaramillo, R. Gomperts, R. E. Stratmann, O. Yazyev, A. J. Austin, R. Cammi, C. Pomelli, J. W. Ochterski, R. L. Martin, K. Morokuma, V. G. Zakrzewski, G. A. Voth, P. Salvador, J. J. Dannenberg, S. Dapprich, A. D. Daniels, O. Farkas, J. B. Foresman, J. V. Ortiz, J. Cioslowski, and D. J. Fox, Gaussian, Inc., Wallingford CT, 2010.
